# Fibroids in Pregnancy: A Case Series

**DOI:** 10.7759/cureus.87577

**Published:** 2025-07-09

**Authors:** Sofia Amber, Asma Fahad, Sadia Maqbool, Nighat Aftab, Saima Faraz

**Affiliations:** 1 Obstetrics and Gynaecology, Latifa Women and Children Hospital, Dubai, ARE; 2 Obstetrics and Gynaecology, Dubai Academic Health Corporation, Dubai, ARE; 3 Obstetrics and Gynaecology, Kings College Hospital, Dubai, ARE

**Keywords:** case report, fetal outcomes, fibroid, obgy (obstetrics and gynecology), pregnancy

## Abstract

Uterine fibroids are common benign tumors that can complicate pregnancy by affecting maternal and fetal outcomes. The impact depends on fibroid size, number, and location. This case series aims to highlight clinical outcomes in pregnancies complicated by fibroids. Three pregnant women with known uterine fibroids were followed through antenatal, intrapartum, and postpartum periods at a tertiary care hospital. Maternal demographics, fibroid characteristics, pregnancy complications, mode of delivery, and neonatal outcomes were reviewed. All patients conceived spontaneously and were diagnosed with fibroids between early pregnancy and the second trimester. Fibroid sites included the cervix, anterior lower uterine segment, and anterior/posterior subserosal regions, with sizes ranging from 4.4×4.5 cm to 12×11.2 cm. All deliveries were conducted by lower-segment cesarean section between 35 and 36 weeks of gestation. Two neonates required NICU admission for respiratory distress, though no cases of birth asphyxia were reported. Intraoperative difficulties were encountered mainly in cases with cervical fibroids. Maternal outcomes were favorable with no major postoperative complications. It is concluded that fibroids in pregnancy, particularly those that are large or lower-segment located, increase the risk of preterm birth and cesarean delivery. Careful antenatal monitoring and individualized delivery planning are essential to optimize maternal and neonatal outcomes.

## Introduction

Uterine fibroids, or leiomyomas, represent the most common benign tumors of the female reproductive system. These smooth muscle neoplasms arise from the myometrium and are typically classified based on their location within the uterus as submucosal, intramural, or subserosal. Their occurrence in pregnancy is not uncommon, with estimates suggesting that 1 in 10 pregnant women may have fibroids [1]. However, due to the asymptomatic nature of many fibroids, their true prevalence may be underreported. Advances in imaging technologies, especially transvaginal and transabdominal ultrasound, have improved the accuracy of detecting fibroids early in pregnancy. The growth of fibroids during pregnancy is influenced by hormonal changes, particularly the increased levels of estrogen and progesterone [2]. While some fibroids may remain stable or even decrease in size, others can undergo rapid enlargement. Studies have shown that growth tends to be more pronounced in the first trimester and often plateaus or regresses in the later stages [3]. The vascular supply to the fibroid and the presence of pregnancy-induced changes, such as red degeneration (a type of fibroid degeneration characterized by hemorrhagic infarction), can cause acute pain and may mimic other obstetric emergencies such as placental abruption [4].

Most pregnant women with fibroids do not experience significant complications. However, when complications do arise, they can impact both maternal and fetal health. Common maternal complications include severe pain, increased risk of miscarriage, bleeding during early pregnancy, and preterm labor [5]. For the fetus, fibroids can lead to malpresentations such as breech or transverse lie, intrauterine growth restriction, and rarely, fetal demise. Larger fibroids, especially those located in the lower uterine segment, may obstruct labor, necessitating cesarean delivery [6]. Furthermore, fibroids can distort the uterine cavity, interfering with normal placental implantation and increasing the risk of placenta previa or abruption. The diagnosis of fibroids during pregnancy primarily relies on ultrasound imaging. MRI may be employed in select cases when the diagnosis is uncertain or if complex anatomical relationships need further clarification, as it provides superior soft tissue contrast without ionizing radiation, making it safe for the fetus after the first trimester [7]. Management of fibroids in pregnancy is generally conservative. Treatment focuses on symptom relief, mainly using analgesics for pain management. Surgical intervention, such as myomectomy, is rarely performed during pregnancy due to the high risk of hemorrhage and the potential for pregnancy loss [8]. Exceptions include cases of intractable pain from torsed pedunculated fibroids or significant obstruction causing urinary retention. In most cases, myomectomy is deferred until the postpartum period if necessary. Careful planning is required for delivery, particularly in cases with large or strategically located fibroids, where cesarean section might be the safer option [9].

## Case presentation

Case 1

**Figure 1:**
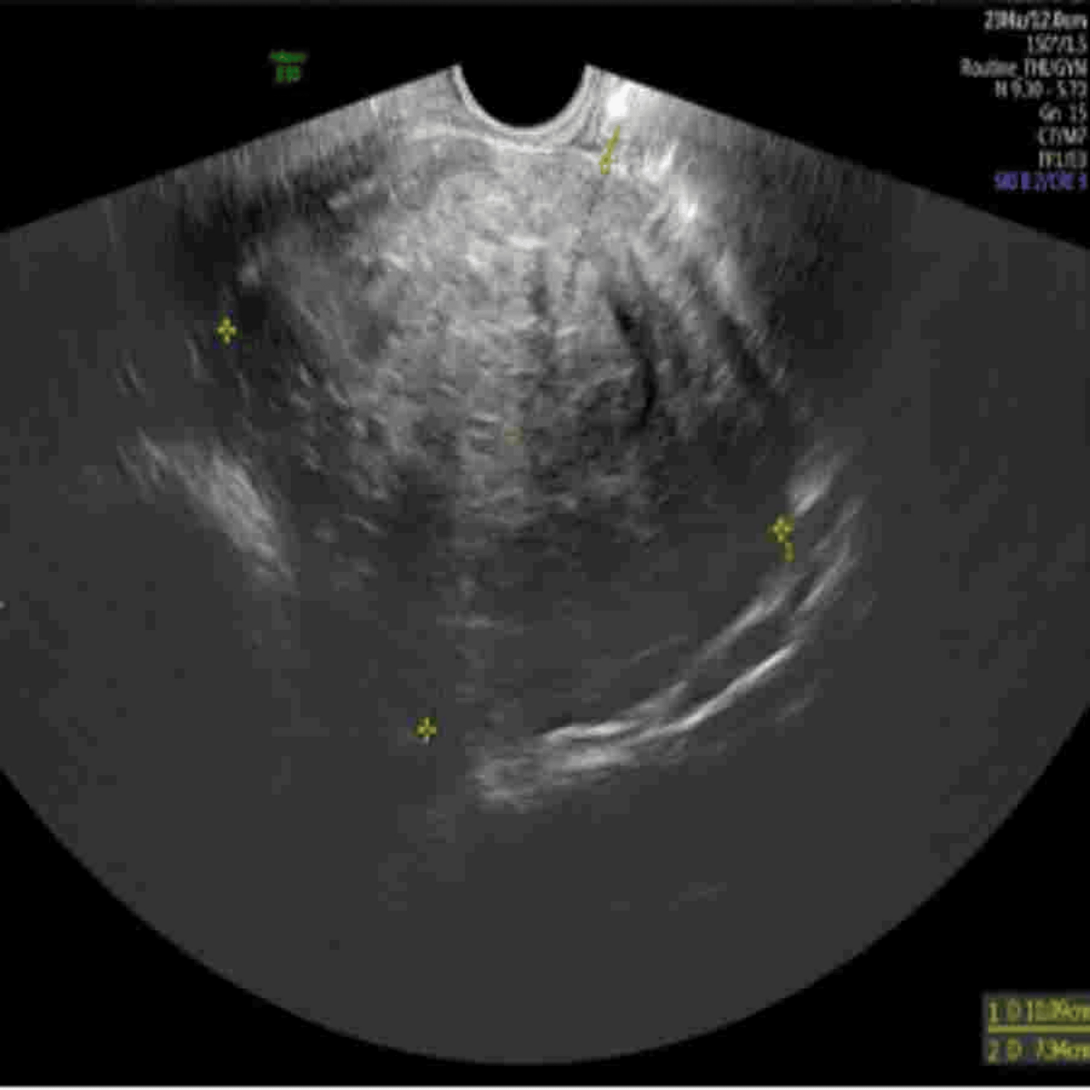
Transvaginal ultrasound showing a large hypoechoic cervical fibroid measuring approximately 10.09×7.94 cm, distorting the lower uterine segment and obscuring visualization of the internal cervical os.

A 36-year-old primigravida woman from the Philippines, with a spontaneous conception, presented to the ED at 36 weeks of gestation with complaints of fluid leakage per vagina. She had no significant past medical or surgical history. An early pregnancy ultrasound at 11 weeks revealed a large posterior lower-segment uterine fibroid measuring approximately 8 cm in diameter. At presentation, a transabdominal ultrasound was performed, which showed anhydramnios (no measurable amniotic fluid pockets) and an anterior placenta, with its lower edge positioned 2.5 cm above the internal cervical os. A large cervical fibroid measuring 10.1×7.9 cm was identified, located low in the uterus, completely obscuring the internal os. Due to this obstructive lesion, the cervix was not visualized on imaging. The patient was admitted to the labor ward for close observation. Shortly thereafter, she developed antepartum hemorrhage (APH) characterized by a sudden and significant episode of vaginal bleeding. Given the diagnosis of placenta previa, complicated by massive APH and a large cervical myoma obstructing the birth canal, an emergency Category 1 cesarean section was indicated. A midline vertical skin incision was made and later extended superiorly above the umbilicus for optimal exposure. Intraoperatively, a large cervical fibroid was noted occupying the entire pelvis. The uterovesical fold was carefully opened, though the urinary bladder was not visualized due to significant uterine distortion. A transverse incision was made on the lower uterine segment to deliver the baby. The infant was delivered in breech presentation, with clear amniotic fluid, and had Apgar scores of 8 and 9 at 1 and 5 minutes, respectively. The placenta and membranes were delivered intact. Following delivery, the uterine anatomy was noted to be severely distorted. The cervical opening was visualized near the umbilicus. The abdominal incision was extended further, and the uterus, along with the fibroid, was exteriorized. The fibroid was found to have migrated to the fundus of the uterus. An incision was made on the posterior uterine wall, and it was noted that the cervix had detached from the vagina (Figure 1).

Reconstructive Vaginocervical Surgery (Uterus-Preserving)

**Figure 2:**
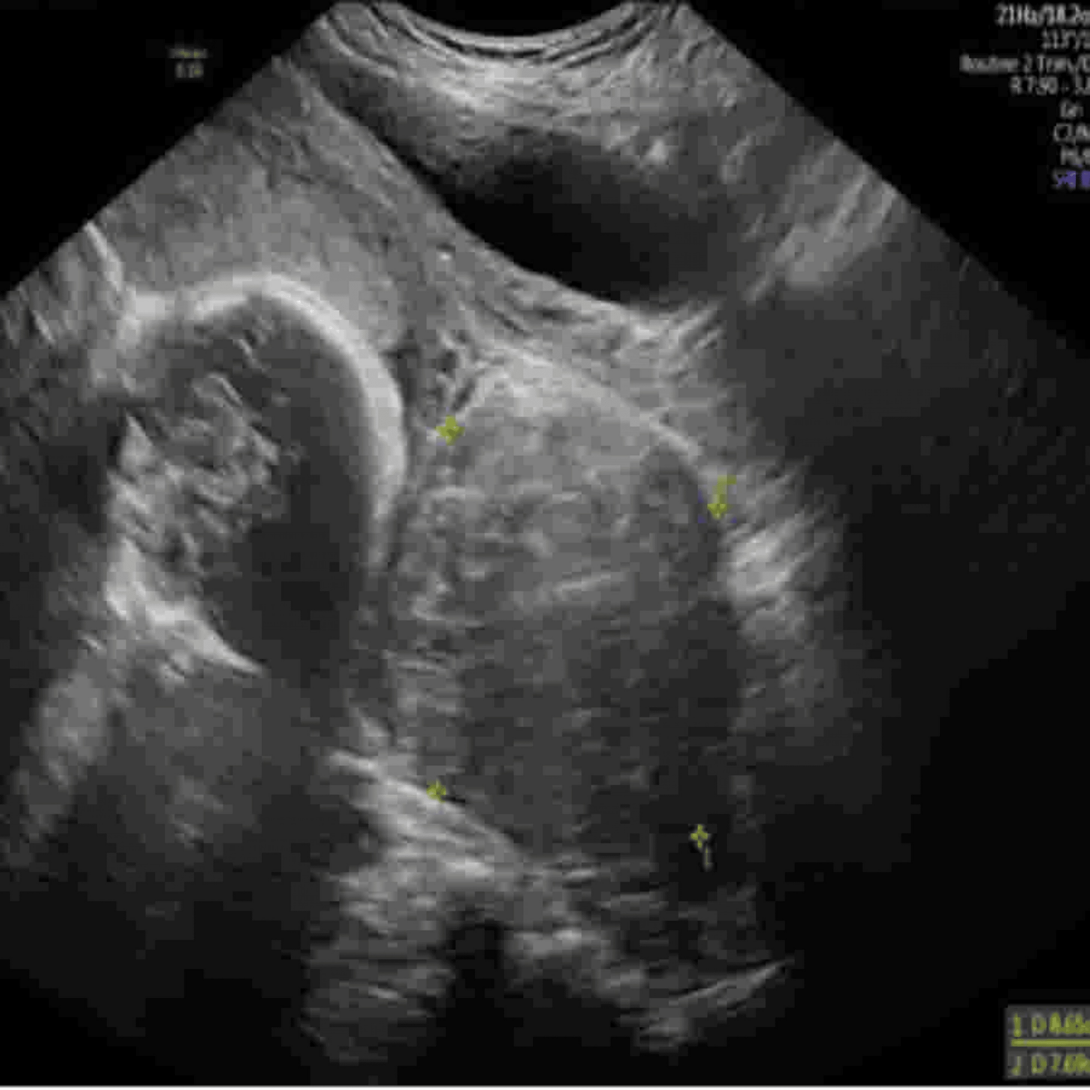
Transabdominal ultrasound showing a large hypoechoic uterine fibroid measuring approximately 8.65×7.69 cm, adjacent to the fetal head, contributing to uterine distortion during the second trimester.

The surgical team proceeded with reconstructive vaginocervical surgery aimed at preserving the uterus. All anatomical landmarks were carefully identified, including the bladder, ovaries, cervix, vagina, uterine arteries, ureters, and round ligaments. A size 18 nasogastric tube was inserted through the endometrial cavity, passing through the cervix into the vagina to assist in alignment during reconstruction. This was removed at the end of the procedure. Multiple reference marks were placed for accurate anatomical alignment. Using 2/0 Vicryl sutures, the vaginal mucosa and fascia were reapproximated to the cervix in interrupted layers, ensuring a two-layer closure. Hemostasis was secured, and good perfusion of all tissues was confirmed with visible vascular pulsations. Surgicel was placed between the posterior bladder wall and the anterior uterine repair site to reduce adhesion formation. The peritoneal layer was closed, and a drain was left in the pouch of Douglas. To support uterine positioning, plication of the round ligaments was performed. The uterosacral and cardinal ligaments were repositioned and anchored appropriately. A final anatomical inspection confirmed a normal pelvic configuration. Urine output was clear, and no injury to the urinary tract was evident. The abdominal incision was closed in layers with suitable suture material. A final vaginal and speculum examination showed no bleeding or visible gaps at the cervix (Figure 2).

Postoperative Course

Intraoperatively, the patient required substantial blood product support to manage ongoing blood loss and maintain hemodynamic stability. A total of seven units of packed RBCs were transfused to address anemia and improve oxygen-carrying capacity. Additionally, four units of fresh frozen plasma were administered to replenish clotting factors and support coagulation. Hemostatic support was further enhanced with the transfusion of three platelet pools to counteract thrombocytopenia and aid in clot formation.

Postoperatively, she was transferred to the ICU for close monitoring over 24 hours. The recovery was uneventful. The patient was discharged in stable condition and was counseled regarding future management. She was advised to undergo laparoscopic myomectomy approximately six months postpartum. For any future pregnancies, elective cesarean delivery was recommended at 32-34 weeks of gestation due to her complex uterine reconstruction and fibroid history.

Case 2

**Figure 3:**
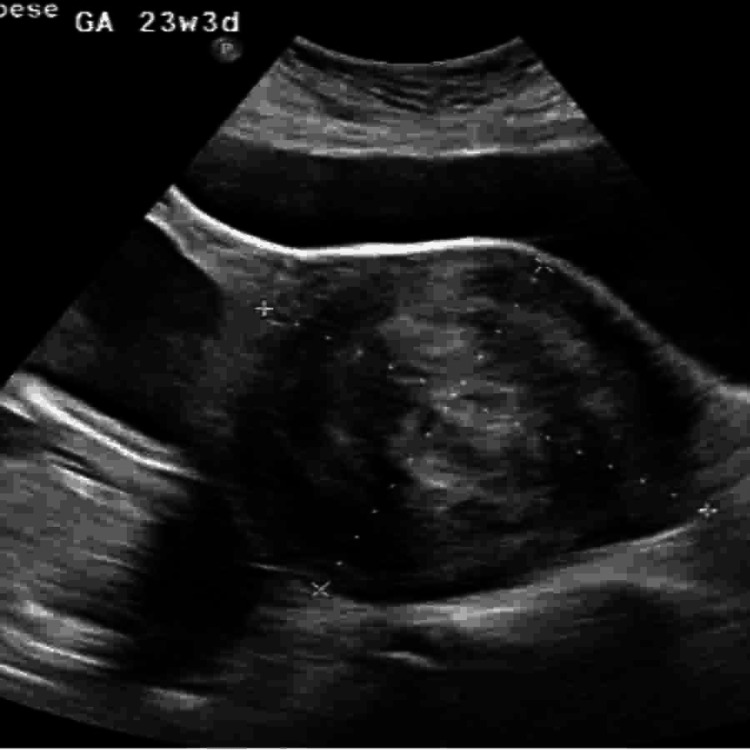
Ultrasound image at 23 weeks and 3 days of gestation showing a large, well-defined hypoechoic uterine fibroid located in the anterior uterine wall. The fibroid measures approximately 11.8×10.6 cm and compresses the adjacent uterine structures, with visible displacement of the fetus.

A 40-year-old Pakistani woman, para 3, with a history of three previous cesarean sections, was booked for antenatal care at 13 weeks of gestation. She had a known history of essential hypertension, managed with labetalol 200 mg twice daily, and type 2 diabetes mellitus, controlled with metformin once daily and insulin Levemir 14 units at night. An anomaly scan performed during the second trimester identified a uterine fibroid located in the lower part of the anterior uterine segment, measuring 12×11.2 cm. The patient continued with regular antenatal follow-up, and due to the history of multiple prior cesarean deliveries and the presence of a significant fibroid, she was scheduled for an elective cesarean section at 36 weeks of gestation. Intraoperatively, the uterus appeared structurally normal, though the lower segment was thickened, consistent with previous surgical history. A large posterior fibroid was noted, occupying the lower uterine region and measuring approximately 16×18 cm. The cesarean section proceeded without complications, and a live baby was delivered in breech presentation, with a birth weight of 2535 g. Bilateral tubal ligation was performed during the same procedure as requested by the patient. The estimated blood loss during surgery was 700 mL. The postoperative course was uneventful, and the patient was discharged in a stable condition (Figure 3).

Case 3

**Figure 4:**
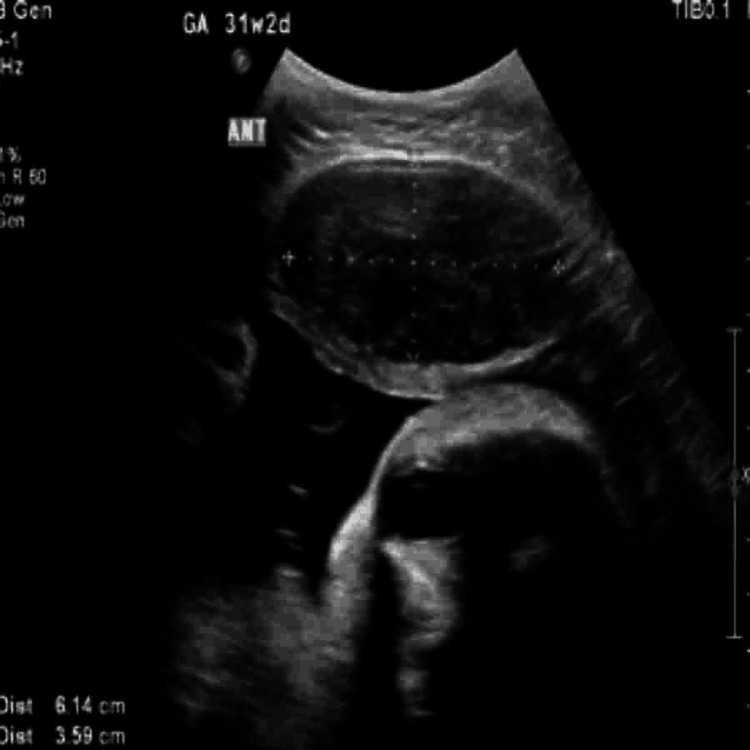
Ultrasound at 31 weeks and 2 days of gestation demonstrating a well-circumscribed hypoechoic anterior uterine wall fibroid measuring approximately 6.14×3.59 cm. The fibroid appears to compress the adjacent uterine structures without directly impinging on the fetus.

**Figure 5:**
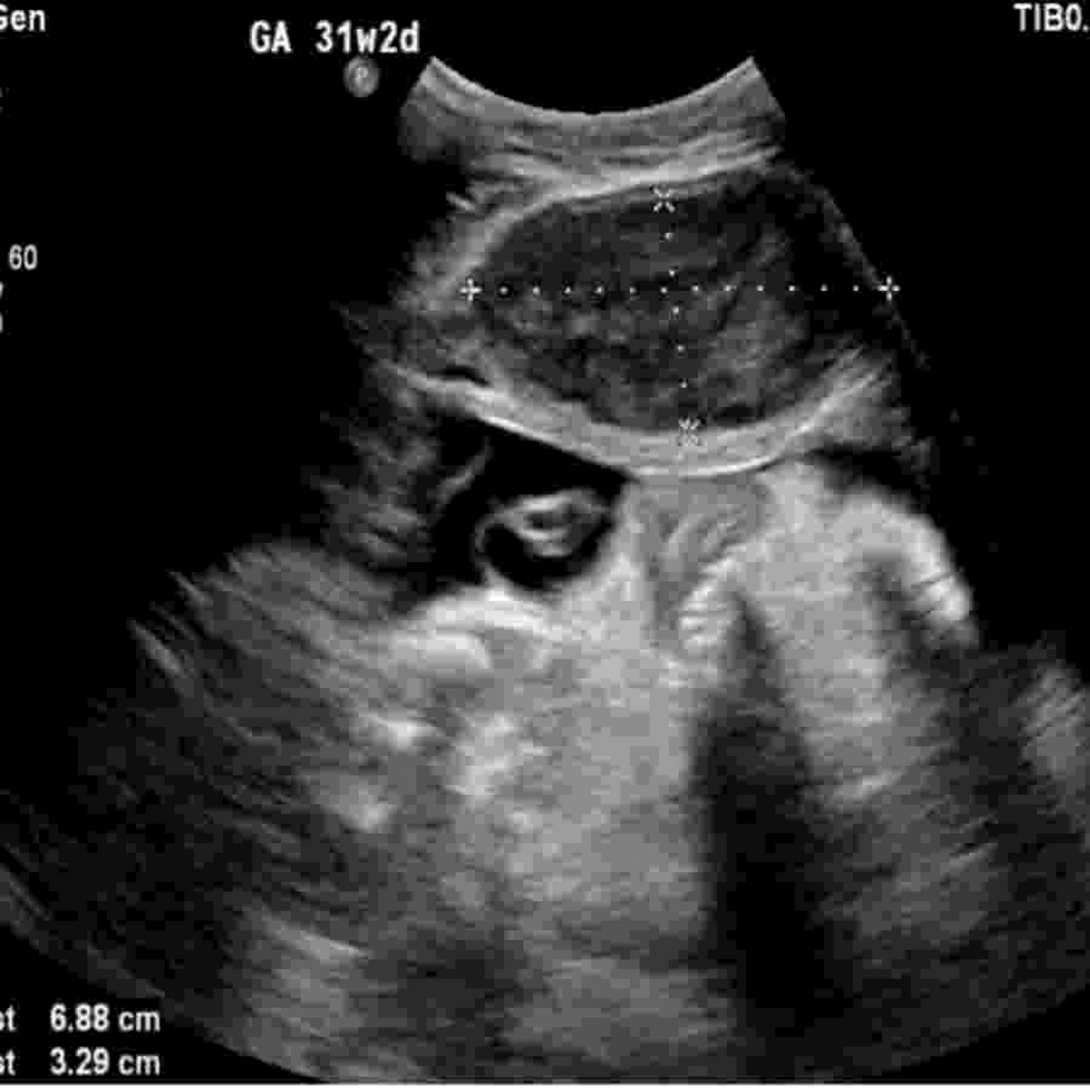
Ultrasound at 31 weeks and 2 days of gestation showing a second anterior uterine wall fibroid measuring approximately 6.88×3.29 cm. The fibroid appears as a well-defined hypoechoic mass, with no immediate compression effects on the fetus, suggesting a stable fibroid during this stage of pregnancy.

**Figure 6:**
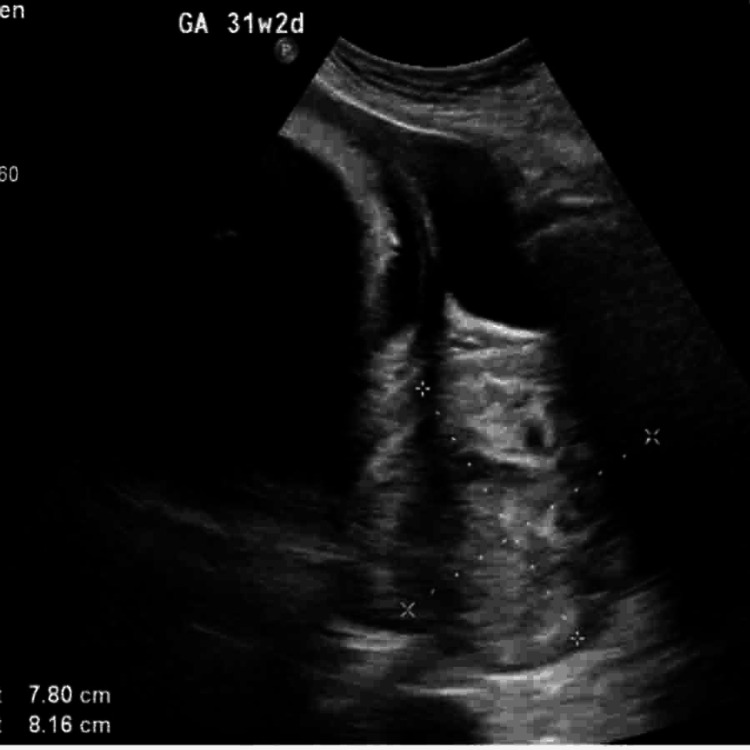
Ultrasound at 31 weeks and 2 days of gestation showing a large cervical fibroid measuring approximately 7.80×8.16 cm. The fibroid appears as a heterogeneous, hypoechoic mass arising from the lower uterine segment, potentially impacting cervical anatomy and influencing delivery planning.

A 35-year-old primigravida woman from Somalia, with a known diagnosis of fibroid uterus, was under regular antenatal follow-up at our hospital. A radiological scan performed at 31 weeks of gestation revealed a large cervical fibroid measuring 7.8×8.2 cm. In addition, two anterior wall myomas were noted, measuring 6.9×3.3 cm and 2.9×2.1 cm, respectively. In view of her diagnosis of a significant cervical fibroid, she was planned for an elective cesarean section at term. However, at 35 weeks of gestation, she presented to the ED in preterm labor and was found to have fetal distress. An emergency cesarean section was therefore performed. Intraoperatively, multiple fibroids were identified. Two large fibroids were noted: one left posterolateral fibroid measuring approximately 7×8 cm and one anterior fibroid measuring approximately 4×4 cm. Additionally, two to three small seedling fibroids were observed anteriorly. Interestingly, no cervical fibroid was visualized during the surgery, despite its previous documentation. The uterus was opened with a transverse incision in the lower uterine segment, just below the anterior wall fibroid. A live baby girl in cephalic presentation was delivered, with clear amniotic fluid drained. The uterine incision was closed in two layers. A small extension of approximately 2 cm was noted in the lower segment near the right uterine angle, which was sutured separately to ensure hemostasis. The estimated intraoperative blood loss was 700 mL. The postoperative period was uneventful, and the patient recovered well. She was advised to undergo an MRI study six months after delivery to reassess the uterine fibroids and guide future management (Figures 4-6).

Summary of findings

The study included three patients with fibroids complicating pregnancy, all conceived spontaneously. The maternal age ranged from 35 to 40 years. Fibroids were located in the cervical, lower anterior uterine segment and anterior/posterior subserosal regions, with sizes ranging from 4.4×4.5 cm to 12×11.2 cm. All patients delivered preterm by lower segment cesarean section (LSCS) , and two neonates required NICU admission (Table 1).

**Table 1:** Summary of maternal, fibroid, and perinatal outcomes GA, gestational age; LSCS, lower segment cesarean section

S. no	Age (years)	Parity	Conception	Fibroid site	Fibroid size (cm)	GA at delivery	Mode of delivery	Birth weight (g)	NICU admission
1	36	P0+0	Spontaneous	Cervical	10.1×7.9	36w1d	LSCS	2140	Yes
2	40	P3+0	Spontaneous	Lower anterior uterine segment	12×11.2	36w0d	LSCS	2535	No
3	35	P0+0	Spontaneous	Anterior and posterior subserous	4.4×4.5; 8.3×6.3	35w6d	LSCS	2530	Yes

## Discussion

Uterine fibroids are the most common benign tumors in women of reproductive age, and their presence during pregnancy can significantly complicate maternal and perinatal outcomes. This case series evaluated three patients with fibroid-associated pregnancies, highlighting the antenatal, intrapartum, and postpartum challenges encountered. All patients in this study conceived spontaneously and were diagnosed with fibroids either in early pregnancy or during the second trimester [10]. Fibroid locations varied, including cervical, anterior lower uterine segment, and combined anterior-posterior subserosal sites. Fibroid size ranged from 4.4×4.5 cm to 12×11.2 cm, with the cervical fibroid posing the greatest technical difficulty during delivery. Consistent with the existing literature, larger fibroids and those located in the lower uterine segments were associated with increased obstetric interventions [11].

All deliveries occurred preterm between 35 and 36 weeks of gestation, and all were performed by elective or emergency LSCS. Cesarean delivery was necessitated by factors such as fetal malpresentation, obstructed labor risk, or fetal distress, aligning with previous studies reporting increased cesarean rates in fibroid pregnancies [12]. Intraoperative challenges included technical difficulties due to fibroid location, and in one case, postpartum hemorrhage was observed. Postnatally, one patient required a blood transfusion, and another experienced uterine subinvolution, though no sepsis or severe morbidity was noted. Two neonates required NICU admission, mainly due to respiratory complications related to prematurity rather than direct fibroid effects. Birth weights varied from 2140 to 2535 g, and no cases of birth asphyxia were observed [13].

The impact of fibroid characteristics such as size, number, and location appears pivotal in determining pregnancy outcomes. Large fibroids (>5 cm), cervical or lower uterine fibroids, and multiple fibroids were associated with more adverse maternal and neonatal outcomes. Conservative management, with careful antenatal monitoring, was key to optimizing outcomes, although surgical interventions such as myomectomy were deferred postpartum in all cases [14]. This study's findings are consistent with previous reports emphasizing the need for individualized delivery planning in women with fibroids [15]. Early diagnosis, multidisciplinary team involvement, and planned cesarean at an optimal gestational age remain critical components of care. Further larger studies are needed to refine guidelines regarding the timing of delivery and surgical strategies for fibroids detected during pregnancy. This study has several limitations. The main limitation of this study is the small sample size, which restricts the generalizability of the findings to a broader population. As a retrospective case series, it may also be subject to selection bias and incomplete data recording. Additionally, the absence of long-term postpartum follow-up limits the assessment of fibroid regression, recurrence, or subsequent fertility outcomes. Imaging modalities were primarily ultrasound-based without standardized MRI confirmation for all cases, which may have affected the detailed characterization of fibroid features.

## Conclusions

It is concluded that the presence of uterine fibroids during pregnancy can significantly impact maternal and perinatal outcomes, particularly when the fibroids are large, multiple, or located in the lower uterine segment or cervix. Careful antenatal monitoring, individualized delivery planning, and readiness for cesarean section are essential for optimizing outcomes. In this case series, all patients required cesarean delivery, and prematurity was a common finding, although major maternal or neonatal morbidity was minimized with timely intervention.
